# Equity in the recovery of elective and oncological surgery volumes after the COVID-19 lockdown: a multicentre cohort study in Italy

**DOI:** 10.1186/s12939-024-02127-1

**Published:** 2024-03-15

**Authors:** Chiara Di Girolamo, Roberta Onorati, Tania Landriscina, Roberto Gnavi, Giulia Cesaroni, Enrico Calandrini, Lucia Bisceglia, Caterina Fanizza, Teresa Spadea

**Affiliations:** 1 Department of Clinical and Biological Sciences, University of Turin, Regione Gonzole, 10, Orbassano (Turin), Italy; 2 Regional Health and Social Care Agency Emilia-Romagna Region, Viale Aldo Moro, 21, Bologna, Italy; 3 Epidemiology Unit, ASL TO3 Piedmont Region, Viale Sabaudia, 164, Grugliasco (Turin), Italy; 4 Department of Epidemiology, Regional Health Service Lazio, Via Cristoforo Colombo, Rome, 112 Italy; 5 Regional Healthcare Agency of Puglia Region, Lungomare Nazario Sauro, Bari, 33 Italy

**Keywords:** COVID-19, Social inequalities, Surgery, Italy

## Abstract

**Background:**

The COVID-19 pandemic has had, and still has, a profound impact on national health systems, altering trajectories of care and exacerbating existing inequalities in health. Postponement of surgeries and cancellation of elective surgical procedures have been reported worldwide. In Italy, the lock-down measures following the COVID-19 pandemic caused cancellations of surgical procedures and important backlogs; little is known about potential social inequalities in the recovery process that occurred during the post-lockdown period. This study aims at evaluating whether all population social strata benefited equally from the surgical volumes’ recovery in four large Italian regions.

**Methods:**

This multicentre cohort study covers a population of approximately 11 million people. To assess if social inequalities exist in the recovery of eight indicators of elective and oncological surgery, we estimated Risk Ratios (RR) through Poisson models, comparing the incidence proportions of events recorded during COVID-19 (2020-21) with those in pre-pandemic years (2018-19) for each pandemic period and educational level.

**Results:**

Compared to 2018-19, volumes of elective surgery showed a U-shape with the most significant drops during the second wave or the vaccination phase. The recovery was socially unequal. At the end of 2021, incidence proportions among highly educated people generally exceeded the expected ones; RRs were 1.31 (95%CI 1.21–1.42), 1.24 (95%CI 1.17–1.23), 1.17 (95%CI 1.08–1.26) for knee and hip replacement and prostatic surgery, respectively. Among low educated patients, RR remained always < 1. Oncological surgery indicators showed a similar social gradient. Whereas volumes were preserved among the highly educated, the low educated were still lagging behind at the end of 2021.

**Conclusions:**

Surgical procedures generally returned to pre-pandemic levels but the low educated experienced the slowest recovery. An equity-oriented appraisal of trends in healthcare provision should be included in pandemic preparedness plans, to ensure that social inequalities are promptly recognised and tackled.

**Supplementary Information:**

The online version contains supplementary material available at 10.1186/s12939-024-02127-1.

## Background

Since the inception of the COVID-19 pandemic, almost 26 000 000 cases have been recorded and more than 190 000 deaths have occurred in Italy [1]. Besides its effects on people’s health, the pandemic has had, and still has, a profound impact on the national health system, altering trajectories of care and exacerbating existing inequalities in health [2]. Postponement of surgeries and cancellation of elective surgical procedures were public health early responses to mitigate the spread of the infection in patients and health care professionals [3]. In Italy, during the first phases of the pandemic, elective surgeries, such as hip and knee replacement, plunged dramatically [4] and people in low socioeconomic positions were more affected by the cut in surgical volumes [2].

There is evidence that elective surgeries postponements and cancellations resulted in an important backlog, although national data for 2021 showed that some procedures returned to pre-pandemic levels [5, 6]. However, as highlighted by the World Health Organization, this recovery, which may help to prevent a further grow of the backlog, may not be enough to reduce it [7]. Moreover, little is known about which population social strata are lagging behind in the recovery process and have suffered the most from the backlog resulting from the reduction of surgical activities.

Therefore, this study aims at evaluating whether, after the 2020 COVID-19 lockdown and throughout 2021, all population social strata benefited equally from the recovery of elective and oncological surgical volumes in four Italian regions: Piedmont, Emilia-Romagna, Lazio, and Puglia.

## Materials and methods

### Study design, population, and data sources

This is a multicentre retrospective study with a closed cohort approach, carried out within the MIMICO-19 network [4] and based on the individual record linkage of regional health administrative and statistical data sources via a unique anonymous key [8].

The study population was derived from the health population registers and the last census held in 2011. It consisted of the residents as of 1 January 2018 in Piedmont, Emilia-Romagna, Lazio, and Puglia, aged ≥ 30 years at the 2011 census and still alive during the observation time. The census was the source of information on the socioeconomic position (SEP) measured through the educational level in adulthood (i.e., in those aged ≥ 30 years at census). Hospital discharge archives were used to retrieve the outcomes of interest within the cohort from January 2018 to December 2021 (hospital discharge data for Piedmont were available until July 2021). The average surgical procedures carried out in the years 2018-19 were considered the expected volumes (pre-pandemic period) and were compared with the observed volumes from the inception of the COVID-19 pandemic (1st March 2020) until the end of 2021 (pandemic period).

### Outcomes

We chose eight indicators of elective surgical volumes encompassing several specialties: total elective surgery, prostatic hyperplasia surgery, laparoscopic cholecystectomy surgery, two indicators of orthopaedic surgery (hip and knee replacement), and three indicators of oncological surgery (lung, colorectal, and female breast cancer). Indicators were based on the definitions of the National Healthcare Outcomes Programme run by the National Agency for Regional Healthcare Services [6]. For each indicator, we included all episodes registered in the study population during the observation periods (Supplemental Table 1 presents the indicators and the International Classification of Diseases, Ninth Revision codes used in their definition).

### Exposures and other variables

Educational level was our SEP indicator and it was chosen because it is stable over time and able to capture socioeconomic conditions from early life to adulthood [9]. It was classified into three levels according to the highest attained qualification: low (primary

education or less, corresponding to the 0–1 levels of the International Standard Classification of Education 1997, modified in 2011 (ISCED-11), middle (lower secondary and short-cycle upper secondary education, ISCED-11: 2–3 C), high (from completed upper secondary onwards, ISCED-11: from 3 A/B upwards) [10].

Age was classified into 5-year age bands (30–34, 35–39,…,85+).

The region corresponds to the region of residence of the subject (Piedmont, Emilia-Romagna, Lazio, Puglia).

We defined six pandemic sub-periods according to the evolution of the pandemic and the introduction of preventive measures: (1) first wave and lockdown (1st March-31st May 2020), (2) summer 2020 (1st June-30th September 2020), (3) second wave (1st October 2020-31st January 2021), (4) population vaccination phase (1st February-30th April 2021), (5) summer 2021 (1st May-31st July 2021), (6) Delta and Omicron spread (1st August-31st December 2021). The first wave and lockdown phase is not the focus of this study and therefore results are only presented in the Supplementary material for comparative purposes.

### Statistical analyses

To evaluate if the post-lockdown recovery has benefitted all educational levels equally, we employed both a descriptive and an analytic approach.

First, for each educational level separately, the volumes of elective surgery in each pandemic sub-period were compared with the expected volumes (average in the corresponding periods of years 2018-19) by means of the percent change [(2020-21–2018-19)/2018-19*100].

Second, we fitted Poisson models adjusted for age, sex, and region, and with an interaction term between educational level and period. Through these models, we estimated the Risk Ratio (RR), which compares the cumulative incidence (or incidence proportion) of events experienced by the study population during the pandemic with those in the pre-pandemic years for each sub-period and educational level.

## Results

The study covers a population of approximately 11 million people, about 26% of the Italian inhabitants aged ≥ 30 years. Lazio contributes for almost 30% to the total population, Piedmont and Emilia-Romagna for about 25% each, and Puglia for 21%. Women accounted for 53% of the total and 56% of the population was aged less than 55 years. The proportion of highly educated people was 42% for men and 39% for women; low educated people were generally older than the middle and highly educated counterparts (Table 1).

**Table 1:** Age, sex, and geographical distribution of the study population by educational level

		Total		Primary or less		Middle school		University degree or high school	
		*N*	col %	*N*	row %	*N*	row %	*N*	row %
**Total**		11 285 253	100	2 653 425	23.5	4 097 864	36.3	4 533 964	40.2
**Age**	30–34	1 027 789	9.1	28 836	2.8	337 307	32.8	661 646	64.4
	35–39	1 301 261	11.5	45 252	3.5	488 195	37.5	767 814	59.0
	40–44	1 387 211	12.3	68 194	4.9	607 045	43.8	711 972	51.3
	45–49	1 385 961	12.3	93 553	6.8	654 606	47.2	637 802	46.0
	50–54	1 205 712	10.7	129 433	10.7	545 210	45.2	531 069	44.0
	55–59	1 074 803	9.5	213 490	19.9	446 661	41.6	414 652	38.6
	60–64	1 067 215	9.5	366 595	34.4	375 798	35.2	324 822	30.4
	65–69	875 398	7.8	414 978	47.4	258 996	29.6	201 424	23.0
	70–74	829 353	7.3	487 390	58.8	197 748	23.8	144 215	17.4
	75–79	598 533	5.3	417 165	69.7	106 179	17.7	75 189	12.6
	≥ 80	532 017	4.7	388 539	73.0	80 119	15.1	63 359	11.9
**Sex**	Men	5 280 434	46.8	988 318	18.7	2 101 313	39.8	2 190 803	41.5
	Women	6 004 819	53.2	1 665 107	27.7	1 996 551	33.2	2 343 161	39.0
**Region**	Piedmont	2 754 385	24.4	641 182	23.3	1 132 173	41.1	981 030	35.6
	Emilia-Romagna	2 835 490	25.1	656 041	23.1	1 043 601	36.8	1 135 848	40.1
	Lazio	3 270 338	29.0	601 211	18.4	1 063 390	32.5	1 605 737	49.1
	Puglia	2 425 040	21.5	754 991	31.1	858 700	35.4	811 349	33.5

Table 2 reports, for each indicator, absolute numbers and percent changes from 2020 to 21 to 2018-19 by pandemic sub-period and educational level.

**Table 2:** Post-lockdown recovery in indicators of elective surgery. Volumes and percent change from each epidemic sub-period (years 2020 and 2021) to expected volumes (average of the corresponding periods in the years 2018 and 2019) by educational level

Indicators	Educational level	June-September			October-January			February-April			May-July			August-December		
		2018-19	2020	Percent change	2018-19	2020-21	Percent change	2018-19	2021	Percent change	2018-19	2021	Percent change	2018-19	2021	Percent change
**Access for elective surgery**	University degree or high school	59 946	56 072	-6.46	74 615	59 189	-20.67	58 390	48 004	-17.79	56 225	50 008	-11.06	63 013	62 912	-0.16
	Middle school	61 640	54 836	-11.04	73 676	55 476	-24.70	58 218	45 271	-22.24	56 631	49 239	-13.05	57 065	55 054	-3.52
	Primary or less	39 181	32 555	-16.91	44 589	30 560	-31.46	35 661	23 955	-32.83	35 780	27 354	-23.55	39 278	32 555	-17.12
	**Total**	**160 767**	**143 463**	**-10.76**	**192 880**	**145 225**	**-24.71**	**152 269**	**117 230**	**-23.01**	**148 635**	**126 601**	**-14.82**	**159 355**	**150 521**	**-5.54**
**Access for knee replacement surgery**	University degree or high school	855	1 170	36.84	1 339	1 263	-5.68	1 021	1 066	4.46	863	1 003	16.22	1 045	1 368	30.91
	Middle school	1 679	2 038	21.42	2 358	2 237	-5.13	1 916	1 828	-4.59	1 681	1 856	10.41	1 718	1 960	14.09
	Primary or less	2 145	2 282	6.39	2 931	2 000	-31.76	2 526	1 600	-36.66	2 210	1 863	-15.68	2 347	2 150	-8.39
	**Total**	**4 679**	**5 490**	**17.35**	**6 628**	**5 500**	**-17.02**	**5 463**	**4 494**	**-17.73**	**4 754**	**4 722**	**-0.66**	**5 110**	**5 478**	**7.20**
**Access for hip replacement surgery**	University degree or high school	1 392	1 707	22.63	2 031	1 840	-9.38	1 591	1 591	0.00	1 347	1 618	20.16	1 601	1 992	24.42
	Middle school	1 799	2 084	15.87	2 388	2 117	-11.33	1 962	1 799	-8.28	1 802	1 970	9.32	1 721	2 087	21.30
	Primary or less	1 914	2 023	5.72	2 554	1 892	-25.92	2 079	1 503	-27.71	1 891	1 749	-7.51	2 149	2 077	-3.35
	**Total**	**5 104**	**5 814**	**13.91**	**6 972**	**5 849**	**-16.11**	**5 632**	**4 893**	**-13.11**	**5 040**	**5 337**	**5.90**	**5 471**	**6 156**	**12.53**
**Access for prostatic hyperplasia surgery**	University degree or high school	1 083	1 245	15.01	1 526	1 209	-20.77	1 200	918	-23.47	1 061	1 082	2.03	1 192	1 394	16.95
	Middle school	1 109	1 095	-1.26	1 351	964	-28.65	1 073	752	-29.88	1 037	939	-9.41	954	1 051	10.17
	Primary or less	709	604	-14.81	853	500	-41.35	707	332	-53.04	655	435	-33.54	666	538	-19.22
	**Total**	**2 901**	**2 944**	**1.50**	**3 730**	**2 673**	**-28.33**	**2 979**	**2 002**	**-32.80**	**2 752**	**2 456**	**-10.74**	**2 812**	**2 983**	**6.08**
**Access for laparoscopic cholecystectomy surgery**	University degree or high school	2 095	1 942	-7.30	2 632	1 762	-33.05	2 075	1 495	-27.95	1 982	1 686	-14.91	2 281	2 224	-2.50
	Middle school	2 241	1 838	-17.98	2 573	1 629	-36.68	2 026	1 301	-35.78	2 083	1 574	-24.44	2 143	2 067	-3.55
	Primary or less	1 233	1 006	-18.41	1 399	830	-40.65	1 100	625	-43.16	1 126	744	-33.90	1 295	1 194	-7.76
	**Total**	**5 569**	**4 786**	**-14.06**	**6 603**	**4 221**	**-36.07**	**5 201**	**3 421**	**-34.22**	**5 190**	**4 004**	**-22.85**	**5 719**	**5 485**	**-4.08**
**Access for malignant breast cancer surgery**	University degree or high school	2 134	1 803	-15.51	2 095	2 201	5.06	1 579	1 725	9.28	1 686	1 764	4.63	2 042	2 246	9.99
	Middle school	1 775	1 378	-22.34	1 763	1 809	2.64	1 334	1 275	-4.39	1 429	1 414	-1.05	1 548	1 626	5.04
	Primary or less	1 242	1 023	-17.63	1 258	1 070	-14.94	1 006	763	-24.12	981	897	-8.52	1 257	1 156	-8.04
	**Total**	**5 151**	**4 204**	**-18.38**	**5 116**	**5 080**	**-0.69**	**3 918**	**3 763**	**-3.94**	**4 096**	**4 075**	**-0.50**	**4 847**	**5 028**	**3.73**
**Access for malignant lung cancer surgery**	University degree or high school	352	389	10.51	370	369	-0.27	278	298	7.39	289	344	19.03	385	400	4.03
	Middle school	436	415	-4.82	450	449	-0.22	323	335	3.88	368	327	-11.14	449	427	-4.90
	Primary or less	375	285	-23.90	337	307	-8.77	274	215	-21.53	296	227	-23.18	373	297	-20.38
	**Total**	**1 163**	**1 089**	**-6.32**	**1 157**	**1 125**	**-2.72**	**874**	**848**	**-2.97**	**953**	**898**	**-5.72**	**1 207**	**1 124**	**-6.84**
**Access for malignant colorectal cancer surgery**	University degree or high school	677	571	-15.59	650	568	-12.62	487	484	-0.62	550	553	0.64	647	728	12.61
	Middle school	780	647	-17.00	767	696	-9.20	555	571	2.98	607	601	-0.99	706	688	-2.55
	Primary or less	938	737	-21.43	816	734	-9.99	655	569	-13.13	715	593	-17.06	912	811	-11.03
	**Total**	**2 394**	**1 955**	**-18.34**	**2 232**	**1 998**	**-10.48**	**1 697**	**1 624**	**-4.27**	**1 872**	**1 747**	**-6.65**	**2 264**	**2 227**	**-1.63**

After a slight recovery during the 2020 summer months following the first lockdown (March-May 2020– Supplemental Fig. 1), volumes of orthopaedic, prostatic hyperplasia, and laparoscopic cholecystectomy surgery, procedures that are likely to be deferrable, dropped again during the second pandemic wave (October 2020-January 2021) or the population vaccination phase (February-April 2021). Prostatic hyperplasia surgery and laparoscopic cholecystectomy surgery showed the largest declines relative to the comparison periods in 2018-19 (-32.8% during the population vaccination phase and − 36.7% during the second pandemic wave, respectively). Oncological procedures underwent smaller average reductions both during the lockdown and afterwards, although breast and colorectal cancer surgery fell up to one fourth during the 2020 summer months (June-September). However big, volume contractions never reached the negative peaks registered during the first lockdown (Supplemental Fig. 1). During the last observation period (August-December 2021), all indicators but total elective surgery, laparoscopic cholecystectomy, lung and colorectal cancers surgery (i.e., knee and hip replacement surgery, prostatic hyperplasia and breast cancer surgery) returned to pre-pandemic levels (Table 2).

With few exceptions, a clear indirect educational gradient was evident for all indicators and across all observation periods. Low educated people showed negative percent changes most of the time (largest negative reduction occurring for prostatic hyperplasia surgery during the population vaccination phase, percent change: -53.04%) whereas among the highly educated, surgical procedures carried out during the pandemic period sometimes outnumbered those registered in 2018-19 (largest gain occurring for knee replacement surgery during summer 2020, percent change: 36.84%).

**Fig. 1:**
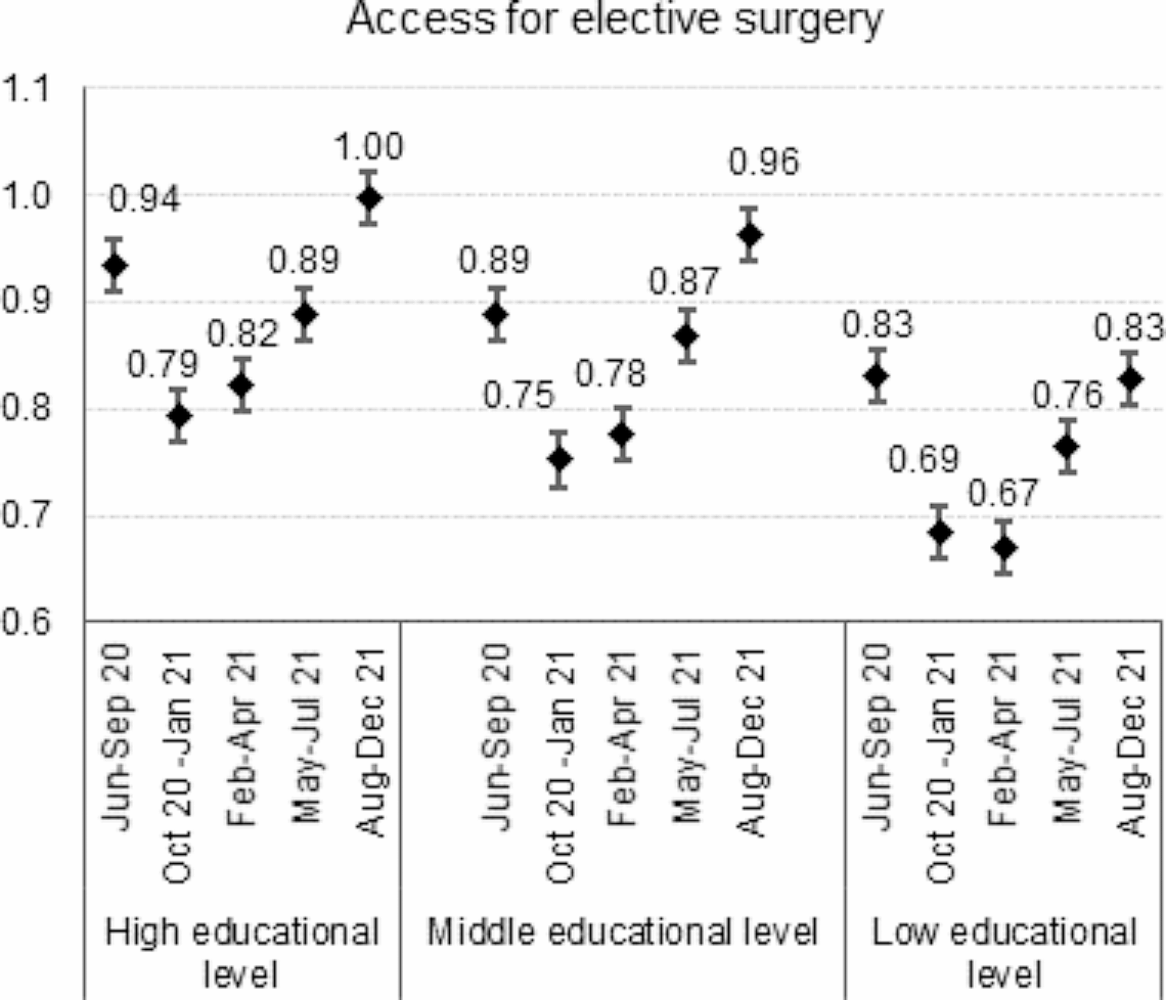
Post-lockdown recovery in total elective surgery. Legend: Risk Ratios and 95% confidence intervals comparing the 2020-21 volumes to the 2018-19 average volumes adjusted for age by epidemic sub-period and educational level

**Fig. 2:**
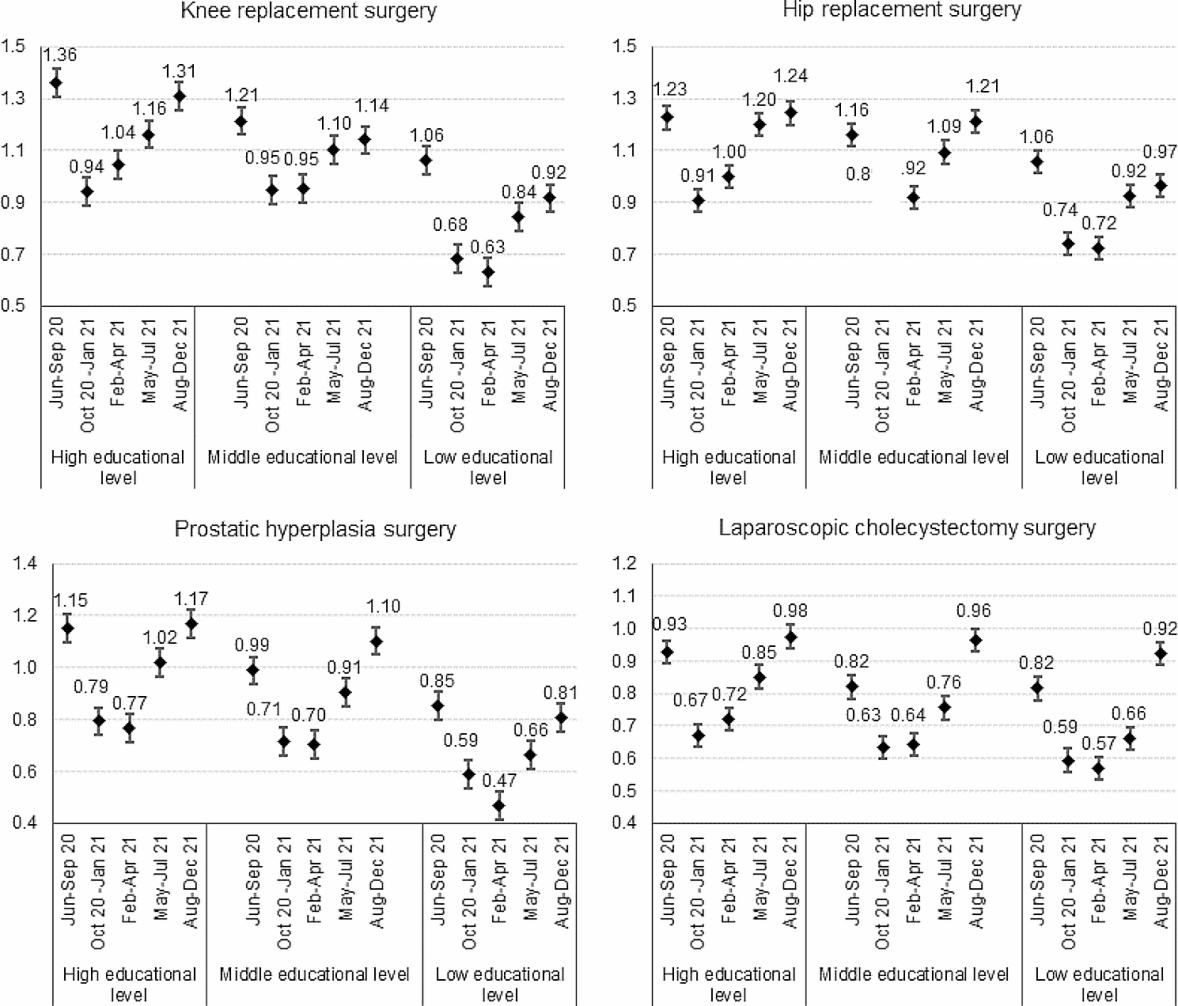
Post-lockdown recovery in orthopedic, prostatic hyperplasia, and laparoscopic cholecystectomy surgery. Legend: Risk Ratios and 95% confidence intervals comparing the 2020-21 volumes to the 2018-19 average volumes adjusted for age by epidemic sub-period and educational level

The age and sex-adjusted risk ratios comparing the five epidemic phases to the 2018-19 corresponding periods for total elective surgery and the orthopaedic, prostatic hyperplasia, and laparoscopic cholecystectomy surgery present a U-shape with the lowest point estimates being recorded during the second wave or the population vaccination phase (Figs. 1 and 2).

As the pandemic unfolded over time, volumes of these procedures progressively increased but at a speed that was differential between the social strata. At the end of 2021, the observed incidence proportions among middle and, even more, highly educated people exceeded the expected ones for all indicators but laparoscopic cholecystectomy. RR were 1.14 (95%CI 1.07–1.22) and 1.31 (95%CI 1.21–1.42) for knee replacement, 1.21 (95%CI 1.14–1.29) and 1.24 (95%CI 1.17–1.23) for hip replacement, 1.10 (95%CI 1.01–1.20) and 1.17 (95%CI 1.08–1.26) for prostatic hyperplasia surgery, among the middle and highly educated subjects, respectively. Low educated patients experienced the greatest volume contractions and, although an upward trend after the second wave was still visible, risk ratios always remained smaller than 1, suggesting that surgical volumes never returned to pre-pandemic levels.

**Fig. 3:**
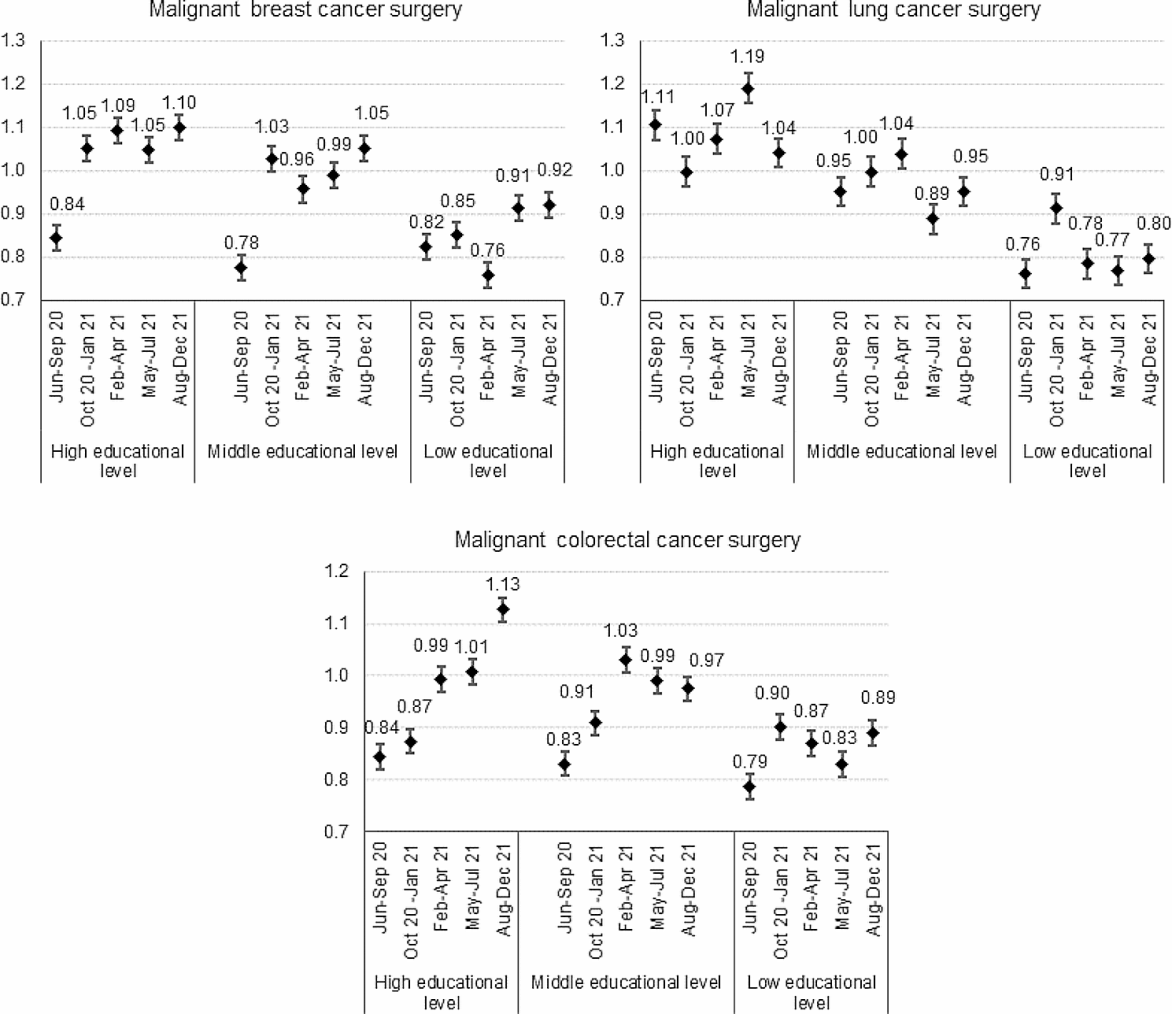
Post-lockdown recovery in oncological surgery. Legend: Risk Ratios and 95% confidence intervals comparing the 2020-21 volumes to the 2018-19 average volumes adjusted for age by epidemic sub-period and educational level

Indicators of oncological surgery showed a less defined evolution over time, but a similar social gradient. The adjusted risk ratios generally revealed that, despite the pandemic, volumes of surgery were preserved among patients with higher educational degrees (Fig. 3).

On the contrary, the low educated paid almost entirely the toll experiencing the greatest reductions and still lagging behind in the recovery at the end of 2021 (female breast cancer RR: 0.92, 95%CI 0.85-1.00, lung cancer RR: 0.80, 95%CI 0.68–0.93, colorectal cancer RR: 0.89, 95%CI 0.81–0.98).

## Discussion

### Summary of main findings

Compared to 2018-19, volumes of total elective surgical procedures and orthopaedic, prostatic hyperplasia, and laparoscopic cholecystectomy surgery showed a U-shape with the most significant drop recorded during the second wave (October 2020-January 2021) or the population vaccination phase (February-April 2021). The afterward recovery was faster among the highly educated than among the low educated. Among the former, surgical volumes returned to and, at times, outnumbered the pre-pandemic ones, whereas among the latter volumes never returned to the pre-pandemic levels.

Oncological surgery underwent less dramatic average reductions and the overall recovery was less appreciable. However, significant social differences emerged: low educated people paid the highest toll in volume reductions and by the end of 2021 they had not caught up with pre-pandemic levels yet.

### Interpretation and comparison with other studies

During the early stages of the pandemic, routine hospital services were severely disrupted and elective planned surgeries were cancelled or postponed, resulting in a variety of potential short and mid-term effects on patient care. Early predictions estimated a weekly decrease of 2.4 million elective surgical procedures globally [11]. Real-world data showed that in many European countries elective planned surgery fell during 2020 [12], with drops ranging from 88% during the first wave in Austria to 23% during the second wave in the Netherlands [13, 14]. Curtailments were reported for oncological procedures too, with reductions spanning from 8% during the whole 2020 in the Netherlands to 4% during the second wave in Austria [13, 14]. In Italy, both orthopaedic surgery and oncological procedures plunged during the first wave and throughout 2020 [4, 15, 16] and surgery for fractures of the neck of the femur and hip replacement were still lower than expected at the end of 2021 [17]. The results of this study confirm what previous data have shown and, by extending the follow-up to 2021, provides an up-to-date picture of the mid-term effects of the COVID-19 pandemic on elective planned surgery. At the national level, the sustained contraction of oncological surgery, especially the breast cancer surgery, can be partially explained by the important delays in the organised screening activities caused by the lockdown first and the ongoing COVID-19 emergency later [18]. Additionally, this decrease in the organised screening testing has been reported to be unequal, that is greater among the lower educated and the immigrants [19].

The good news is that volumes of most of the indicators of planned surgery considered in this study came back to pre-pandemic levels. Despite this achievement, the impending surgical backlog resulting from the activity contractions registered throughout 2020 and 2021 remains a critical concern for the National Health System. For example, it has been estimated that nationwide the number of hip replacement, laparoscopic cholecystectomy, and breast cancer surgical interventions dropped by 27,000, 42,000, and 7,800 procedures, respectively during the 2020-21 [6]. As it has been extensively argued [20, 21], cancellations and delays of elective surgical procedures may result in a range of medical consequences affecting patients’ outcomes and wellbeing. Indeed, while the patient awaits surgery, the disease may progress and result in worse outcomes, more morbid operations, more intense and costly treatment, and higher mortality [20]. Two recent meta-analyses quantified the consequences of surgery delays for breast, lung, and colon cancers. Hanna et al. reported a 6–8% increased chance of death for each 4-week delay in surgical treatment [22]. Johnson et al. concluded that a 12-week delay in surgery was associated with decreased overall survival; estimates were larger for stage I and II breast cancer suggesting that survival in these patients may be especially sensitive to surgical delays [23]. Treatment postponement.

has also been associated with deterioration of mental wellbeing and quality of life in cancer patients [24] and in those awaiting orthopaedic surgery in the United States [25, 26].

The bad news is that the recovery of surgical volumes has been socially unequal. Across all the indicators analysed, the most vulnerable strata of the population experienced the greatest contractions and the most modest resumption to pre-pandemic levels. In a previous paper, we reported that during the first seven months of the pandemic, the social gradient in hospital access and volumes, including the surgical ones, became steeper compared to the 2018-19 period [2]. Adding to what was already a worrisome finding, the present study highlights not only that inequalities persist, but also that the pace of recovery has been slower throughout 2021 among the less educated. A slower recovery may be attributed to several reasons, including barriers of access in a still under-pressure health system, patient’s selection and prioritisation by surgical wards, or an actual shrinkage of the at-risk population due to the harvesting effect of COVID-19, which was likely stronger among the more deprived population groups. Social inequalities in the surgical backlog re-entry have been reported elsewhere. According to a study that looked at waiting lists in July 2021 for planned hospital treatment, including knee and hip replacements, people in England’s most deprived areas were 1.8 times more likely to experience a wait of over a year for hospital treatment than those in the most affluent areas [27]. Results for the US return a mixed picture. On the one hand, a study based on the American Society for Clinical Oncology COVID-19 Registry, which followed about 5,000 patients with cancer from April 2020 to September 2022, found that both ethnicity and area-level social determinants of health were associated with cancer treatment delay or discontinuation [28]. On the other hand, Glance et al. found that, among 3 470 905 adults with inpatient hospitalizations for major surgery, the reduction in operations was not differential between White and ethnic minority patients [29].

The important inequities heightened by COVID-19 worldwide represent a public health failure but also an opportunity to rethink and improve surgical care provision, as suggested by the World Health Organization [7]. A multifaceted approach promoting the partnerships between surgeons, primary care professionals, public health experts, and social scientists has been proposed as an effective way forward to tackle inequalities in surgical practice [30]. On a similar note, the Italian Association of Medical Oncology has called for patient-focused and decentralized care as a tool to improve outcomes and quality of life of patients and to reduce costs [31]. Redesigning the organisational models and strengthening the networking between oncologists and other specialists, hospital services, general practitioners, and primary health facilities may optimise patient’s management and contribute to closing the equity gap in cancer care by retaining into the system hard-to-reach and vulnerable populations.

### Strengths and limitations

To the best of our knowledge, this is the first study to assess educational inequalities in surgical volumes throughout the COVID-19 pandemic in Italy, and one of the few in Europe. Moreover, thanks to its extended follow-up, the study allowed us to track the recovery’s pace over time and to assess inequalities trends. The health information and administrative registries sources virtually cover the entire resident population, reducing the risk of selection bias, and allow to efficiently follow people over time and to explore multiple outcomes simultaneously. Moreover, its wide geographical coverage provides a fair approximation of the national situation during the first two years of the COVID-19 pandemic.

The main limitation of the study is that we assumed that 2018-19 was the best comparison time for both 2020 and 2021. This approach, which has been widely used in studies assessing the impact of the pandemic, does not account for the harvesting effect of COVID-19, which, as mentioned before, was likely stronger among the more deprived population groups. Moreover, we could include only those four Italian regions where integrated health and socioeconomic data for the resident population are available through the longitudinal studies. Although these regions are scattered throughout the country and therefore provide a fair approximation of the national picture, it is pivotal to work towards data integration in all the regions in order to have a common and consistent system for monitoring health inequalities on a national scale. Finally, data for Piedmont were not available for the last observation period resulting in a reduction of the statistical power of the study.

## Conclusions

During the first two years of the COVID-19 pandemic, elective and oncological surgical volumes decreased in numbers. Although some procedures returned to pre-pandemic levels, the low educated experienced the biggest drops and the slowest recovery paving the way to an increase in inequalities in surgical treatment.

Ongoing monitoring of local and national trends of healthcare services provided to citizens and the assessment of how different demographic and social groups are performing should be included in pandemic preparedness plans to ensure that arising or enduring social inequalities are promptly recognised and tackled. To such an extent, a nation-wide and up-to-date system of health and social data is urgently needed. Simultaneously, an equity-oriented appraisal, based on tools already available such as the health equity audit, can support service improvement policies implemented at local, ragional, and national levels.

### Electronic supplementary material

Below is the link to the electronic supplementary material.

Supplementary Material 1

## Data Availability

Raw data cannot be made freely available. Aggregated data are available for other researchers upon reasonable request from the corresponding author.

## Abbreviations

ISCED: International Standard Classification of Education
SEP: Socioeconomic position
