# Supplementary material for: Equity in the recovery of elective and oncological surgery volumes after the COVID-19 lockdown: a multicentre cohort study in Italy
Source: Int J Equity Health. 2024 Mar 15;23:57. doi: 10.1186/s12939-024-02127-1 (PMC10943780; doi:10.1186/s12939-024-02127-1)
Supplement: Supplementary file 1 — Supplementary Material 1 [file 12939_2024_2127_MOESM1_ESM.docx]

Supplemental Table 1: list of indicators of surgical volumes with their selection criteria (ICD-9-CM codes).

| **Category** | **Indicators** | **ICD-9-CM diseases codes** |
| --- | --- | --- |
|  |  |  |
| Elective surgery | Access for elective surgery | All elective hospitalizations with surgical DRG |
|  | Access for knee replacement surgery | Procedures: 81.54, 81.55, 00.80, 00.81, 00.82, 00.83, 00.84 |
|  | Access for hip replacement surgery | Procedures: 81.51, 81.52, 81.53, 00.70, 00.71, 00.72, 00.73, 00.85, 00.86, 00.87 |
|  | Access for prostatic hyperplasia surgery | Diseases: 600.xx, 601.xx, 602.0, 602.1, 602.2, 788.2x, 788.4x  Procedures: 60.2x, 60.96, 60.97, men |
|  | Access for laparoscopic cholecystectomy surgery | Procedures: 51.23 |
| Oncological surgery | Access for malignant breast cancer surgery | Diseases: 174.x, 198.81, 233.0 and procedures: 85.2x, 85.33, 85.34, 85.35, 85.36, 85.4x, women |
|  | Access for malignant lung cancer surgery | Diseases: 162.2, 162.3, 162.4, 162.5, 162.8, 162.9, 197.0 and procedures: ICD-9-CM: 32.3, 32.4, 32.5, 32.6, 32.9, 32.29 |
|  | Access for malignant colorectal cancer surgery | Colon: diseases: 153.x, 197.5 and procedures: 45.7x, 45.8, 45.9x, 46.03, 46.04, 46.1x; excluded diseases: 48.49, 48.5, 48.6 Rectum: diseases:154.x, 197.5 and procedures: ICD-9-CM: 48.49, 48.5, 48.6x; excluded procedures: 45.7x, 45.8, 45.9x, 46.03, 46.04, 46.1x |

Supplemental Figure 1: Indicators of elective surgery: percent changes of volumes from 2020 lockdown (March-May) to 2018-19 (average March-May) by educational level.
